# Characterization of Hypervirulent *Klebsiella pneumoniae* (Hv-Kp): Correlation of Virulence with Antimicrobial Susceptibility

**DOI:** 10.1155/2022/4532707

**Published:** 2022-08-18

**Authors:** V. Vandhana, K. Vishwas Saralaya, Sevitha Bhat, Shalini Shenoy Mulki, Archana. K. Bhat

**Affiliations:** Department of Microbiology, Kasturba Medical College, Manipal Academy of Higher Education, Mangalore 575001, Manipal, Karnataka, India

## Abstract

**Introduction:**

Hypervirulent *K. pneumoniae* (Hv-Kp) is an emerging variant of classical *K. pneumoniae* (C-Kp) that exhibits hypermucoviscocity and possesses multiple siderophores as virulence factors and is known to cause serious debilitating infections in immunocompetent individuals. *Aim and objective.* The aim of this study is to identify C-Kp and Hv-Kp strains and detect their virulence factors and antimicrobial susceptibility patterns.

**Materials and Methods:**

A total of 129 *K. pneumoniae* isolates from different clinical samples were used for the identification and differentiation of classical *K. pneumoniae* (C-Kp) and hypervirulent *K. pneumoniae* (Hv-Kp) to correlate their virulence with antimicrobial susceptibility patterns and identify their risk factors. Hypermucoviscosity was determined by a string test (>5 mm of string length). The aerobactin gene was detected by PCR. *Results and Conclusion.* In total, 13.9% (18/129) were Hv-Kp and 86.1% (111/129) were C-Kp. Only 50% (9/18) of the Hv-Kp isolates were hypermucoviscous. C-Kp was significantly more resistant to antimicrobials than Hv-Kp. Among C-Kp, 75.7% were ESBL producers and 76.6% were multidrug resistant while in Hv-Kp, 44.44% were both ESBL producers and multidrug-resistant which is statistically significant (*P* < 0.01). Diabetes was a common risk factor for C-Kp infections whereas, respiratory disorders like COPD and prolonged ICU stay were the risk factors for Hv-Kp infections. The mortality rate among patients with Hv-Kp infections (87.5%) was significantly high when compared to that of C-Kp infections (35.7%) (*P* < 0.001). A majority of hypermucoviscous *K. pneumoniae* isolates were multidrug resistant (65.2%). Although the prevalence of Hv-Kp infections was low, a high percentage of them were multidrug resistant with a significantly high mortality rate. Hence, it is important to efficiently identify Hv-Kp strains from clinical samples and determine their antimicrobial susceptibility patterns, so as to provide immediate and effective treatment and to prevent possible outbreaks.

## 1. Introduction

*Klebsiella pneumoniae* is an opportunistic, Gram-negative enteric bacillus, largely known for causing community acquired and health care associated infections. The two main pathotypes in circulation are the classical *K. pneumoniae* (C-Kp) and hypervirulent *K. pneumoniae* (Hv-Kp) [1].

C-Kp is one among the most prevalent pathogens essentially causing health care associated infections in patients with underlying comorbid conditions, existing barrier breakdown like wound surgeries and in immunocompromised individuals [2]. The ability of this pathotype to acquire multiple elements that confer multidrug resistance including extended spectrum of beta-lactamases (ESBLs) and carbapenemases makes them notorious [2, 3].

Hv-Kp is an emerging variant of C-Kp that exhibits hypermucoviscocity and possesses multiple siderophores as virulence factors and is known to cause serious debilitating infections in immunocompetent individuals [4, 5]. The pathotype has the potential to metastasize from the site of infection to eye, lung, and central nervous system (CNS). The infections associated with hvKp include bacteremia, pneumonia, and soft tissue infections [6].

Hypermucoviscosity was considered a definitive trait of Hv-Kp strains but recent studies have reported nonhypermucoviscous Hv-Kp strains as well as hypermucoviscous C-Kp strains [2, 7]. Recently, a siderophore named aerobactin has been established as a definitive trait of Hv-Kp strains [3]. Aerobactin plays a significant role in virulence as studies have demonstrated its importance in increased iron acquisition by promoting their growth in iron depleted environment like human ascites fluid [8].

The prevalence of antimicrobial resistance is lower in Hv-Kp when compared to C-Kp strains [1]. But recent studies emphasize on the increasing incidence of multidrug resistant Hv-Kp with production of ESBLs and carbapenemases. Studies show that C-Kp strains have acquired the mobilizable Hv-Kp virulence plasmids via horizontal gene transfer and developed into MDR Hv-Kp strains [2, 8]. Diabetes, digestive diseases, surgery, and cancer are found to be the risk factors of Hv-Kp infections [1, 3]. The previous studies published have reported a prevalence of Hv-Kp as 3.7%. [3].

Our study focuses on identifying C-Kp and Hv-Kp strains in our centre and detect their virulence factors, type of infections, and antimicrobial susceptibility patterns.

## 2. Materials and Methods

Study setting: The study was conducted in the Microbiology laboratory of Kasturba Medical College, Mangalore.

The study comprised of all types of clinical samples: blood, urine, pus, tissue, body fluids, swabs, and respiratory samples.

Study design: Prospective study.

Study period: Five months (January 2021- May 2021)

Sampling method: Convenient random sampling.

Sample size: A total of 129  *K. pneumoniae* isolates were collected between January 2021 and February 20211.

Inclusion criteria were as follows: Clinically significant isolates of *Klebsiella pneumoniae* from the mentioned samples that were identified and confirmed either by conventional culture methods and biochemical tests or by the VITEK II Compact system were included.

Exclusion criteria: Duplicates obtained from the same patient, isolates obtained from co-infections, and samples with insufficient clinical data were excluded.

## 3. Methodology

The isolates were identified using the VITEK II compact system as per standard procedure. Confirmed *K. pneumoniae* isolates were stored in 20% glycerol broth at −20°C till use.

String test: Hypermucoviscosity was determined by a string test (>5 mm of string length) [9].

Antibiotic susceptibility testing was performed by an automated VITEK II compact system. The antibiotics tested were piperacillin, ampicillin, amikacin, amoxyclav, gentamicin, fosfomycin, cefotaxime, ceftazidime, cefuroxime, ceftriaxone, ciprofloxacin, norfloxacin, ofloxacin. cotrimoxazole, imipenem, meropenem, ertapenem, tigecycline, piperacillin/tazobactam, amoxycillin/clavulanic acid, and cefaperazone/sulbactam. The results were then interpreted with respect to the 2020 Clinical and Laboratory Standards Institute (CLSI) guidelines [10]. *Escherichia coli* ATCC 25922 was used as quality control for the antimicrobial susceptibility test.

Extended Spectrum of Beta Lactamase (ESBL) detection was done using ceftazidime and ceftazidime combined with clavulanic acid by the Kirby Bauer's disk diffusion method. Difference in the zones of inhibition of more than 6 mm was considered positive for ESBL production [10].

Isolates showing resistance to three or more drugs of different classes were considered multidrug-resistant (MDR). The isolates were considered carbapenem resistant when resistance was observed to either meropenem or imipenem [11].

PCR for aerobactin gene: Aerobactin gene detection was done by conventional PCR [12]. DNA extraction was carried out by boiling method. Aerobactin positive strains were designated as Hv-Kp. Forward and reverse primers for aerobactin gene (556 bp) are 5′GCATAGGCGGATACGAACAT-3′ and 5′-CACAGGGCAATTGCTTACCT-3′, respectively [12].

### 3.1. Statistical Analysis

The clinical data of patients were acquired from medical records including age, sex, clinical presentation, underlying diseases, antimicrobial therapy, and clinical outcomes. All the data were entered in Microsoft Excel sheets and interpreted using appropriate statistical tools in SPSS version 20.0. Correlation of virulence genes, antimicrobial susceptibility, and risk factors among Hv-Kp and C-Kp groups were carried out using Chi square test. A “*P* value”, i.e., a measure of probability of <0.05 was accepted as statistically significant.

## 4. Results

### 4.1. Isolation of *K. pneumoniae* from Various Types of Infections in the Study Population

Urinary tract infections and septicaemia cases were found to yield the highest number of isolates (45/129 and 35/129 isolates, respectively, approximately 62% of total isolates; Table 1). Maximum number of cases (14/129; 10.8%) from which *K. pneumoniae* were isolated was from the male, paediatric age group (<1 year), all from bloodstream infections. This is followed by the male group of age 51–70 years (28/129, 21.7%).

In males and females, 81 and 48 isolates were collected, respectively (Table 1). In females, predictably, the highest number of isolates (26; 20.1%) were from urinary tract infections, indicating the anatomical predisposition for UTI, when compared to males (19, 14.7%; statistically significant, *P* < 0.01).

### 4.2. Differentiation of *K. pneumoniae* into Classical (C-Kp) and Hypervirulent *K. pneumoniae* (Hv-Kp)

Among 129 isolates, 23 isolates (17.8%) were positive for hypermucoviscosity (Table 2). A total of 18 strains (13.9%) were Hv-Kp. Majority of the Hv-Kp strains (14 of 18; 77.7%) were isolated from cases of septicaemia, pyogenic infections, and urinary tract infections.

### 4.3. Determination of Antimicrobial Susceptibility Patterns of C-Kp and Hv-Kp

C-Kp were significantly more resistant to antimicrobials (both in exhibiting multi-drug resistance and ESBL production) than the Hv-Kp isolates (Tables 3 and 4). A significant number of isolates of the C-Kp were found to be of the MDR type and exhibited resistance to at least three classes of antibiotics (76.6% of C-Kp isolates).

It was observed that 75.7% of these strains were flagged as ESBL positive (Table 5). Only a small portion of the C-Kp isolates were found to be sensitive to most of the antibiotics tested and flagged as non-MDR (23.4%). Hv-Kp strains were more commonly found to be of the non-MDR type (55.5%) which was statistically significant when compared to the non-MDR C-Kp isolates (*P* < 0.01). Similarly, there was a significantly lower number of MDR and ESBL + strains among the Hv-Kp isolates (*P* < 0.01; Table 5).

### 4.4. Determination of Risk Factors Associated with Hv-Kp

Records of only 64 cases (56 cases infected with C-Kp and 8 cases of Hv-Kp) could be retrieved which yielded information regarding the outcome of infections of both groups (Table 6). Diabetes was the most common predisposing factor identified in our study in which maximum number of C-Kp (16/56, 28.6%) were isolated, whereas pre-existing respiratory tract diseases such as COPD and intubation/ICU stay were found to predispose to Hv-Kp infections (4/8, 50%; Table 6).The mortality rate among patients infected with Hv-Kp (7/8, 87.5%) was extremely high when compared to the group of patients infected with C-Kp (20/56, 35.7%) and it is statistically significant (*P* < 0.001).

### 4.5. Correlation of Virulence Markers with Antimicrobial Susceptibility Patterns of Hv-Kp

**Figure 1:**
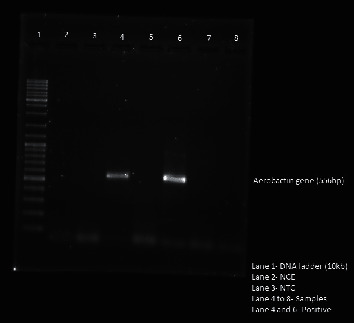
Gel image of detection and amplification (by polymerase chain reaction) of aerobactin gene (556 bp) in *Klebsiella pneumoniae* isolates included in the study. NCE: negative control of extraction, NTC: nontemplate control Hv-*K. pneumoniae*: hypervirulent *Klebsiella pneumoniae,* C- *K. pneumoniae*: classical *Klebsiella pneumoniae,* ESBL+: extended spectrum of beta lactamases positive, MDR: multidrug resistant.

A majority of the hypermucoviscous *K. pneumoniae* isolates were found to be either multidrug resistant (15/23; 65.2%) or ESBL producers (12/23; 52.2%). Only a small percentage of the isolates were non-MDR (8/23; 34.7%). The correlation of the hypermucoviscous phenotype with higher resistance to antibiotics was found to be statistically significant when compared to hypermucoviscosity and non-MDR isolates (*P* < 0.01) (Table 7). The PCR gel picture for the detection of aerobactin gene is shown in Figure 1.

No significant positive correlation was detected between the aerobactin gene and antimicrobial susceptibility patterns of Hv-Kp. Although the number of non-MDR strains possessing the aerobactin gene was slightly higher (55.6% non-MDR strains versus 44.4% MDR strains) than those exhibiting MDR patterns, this difference was not found to be statistically significant Table8.

## 5. Discussion

*Klebsiella pneumoniae* is one of the frequently encountered pathogens that has caught the attention of the clinicians around the world, as it is becoming victorious in causing increasing number of life-threatening infections every year. The bug is associated with community acquired and health care associated infections and is known for multidrug resistance. Our study had focused on understanding the prevalence of Hv-Kp and study their virulence and antimicrobial susceptibility patterns as compared to C-Kp. The isolation rate of *K. pneumoniae* in our study and the supporting studies show an increasing trend and hence has become a major health concern [13, 14].

The majority of the isolates in our study was obtained from the cases of urinary tract infection (UTI) (34.88%). This was followed by septicaemia (27.13%) as observed in a study by Oana Mariana Cristae, et al. [15] *K. pneumoniae* is the third most frequently isolated organism from blood in patients with bacteremia and septicemia.

Our study observed that 51.42% of the total blood culture isolates belonged to the pediatric age group similar to a study conducted by Ramalingam Sekar, et al. [16]. In both male and female groups, the highest number of *K. pneumoniae* associated infections was seen in the older age group of 51–70 years with highest number of cases in males. This could be due to their waning immune system and high chances of comorbidities as noted by other studies [14]. The increased sensitivity of female group to UTI caused by *K. pneumoniae* which is statistically significant compared to the male group can be explained by the anatomical predisposition of the genitourinary system in females.

Our study had considered hypermucoviscosity as a phenotypic feature of Hv-Kp but was only confirmed by the presence of aerobactin gene. A total of 18 isolates (13.95%) were identified as Hv-Kp, considerably low as compared to other studies suggesting the variation in its prevalence in different geographical area [3, 11]. Only 50% of Hv-Kp strains were hypermucoviscous and this observation was similar to the study conducted by Thomas A Russo, et al. [17].

To substantiate the characteristic feature of invasiveness in Hv-Kp, our study has found that maximum number of Hv-Kp strains were isolated from pyogenic infections, bacteremia/septicaemia, and urinary tract infections. The hypermucoviscosity in Hv-Kp isolates also follows the same trend. All the Hv-Kp isolates from pyogenic infection are hypermucoviscous which indicates the role of excessive production of capsular polysaccharide in its invasiveness and metastasis becoming fatal. Isolates from UTI and bloodstream infections alone constituted for 63.96% of the total C-Kp isolates and a similar trend of isolation rate was observed in two other studies [14, 18].

A significantly high percentage of C-Kp isolates (76.6%) in our study showed antibiotic resistance by exhibiting ESBL production and multidrug resistance. Multidrug resistance and ESBL production are comparatively lower in the Hv-Kp group with both 44.44% and is statistically significant (*P* < 0.01). Although this observation agrees with the previous studies, it is comparatively the highest percentage, constraining the fact that they are continuously evolving under the environmental stress created by the misuse of antibiotics and outbreaks of hospital acquired infections [11, 13, 19].

All the ESBL producing isolates of both C-Kp and Hv-Kp were multidrug resistant thus limiting the availability of effective drugs for therapy. CTX—M types of ESBL is more prevalent. ESBL producing C-Kp isolates were found maximum from sepsis and UTI (65.85% collectively). We have found that 35.13% and 16.66% of C-Kp and Hv-Kp isolates were carbapenem resistant respectively. A global spread of chromosomal and plasmid mediated carbapenem resistance genes like *bla*KPC, blaNDM-1, *bla*OXA-48 via horizontal gene transfer, and transduction could be a possible explanation for the emergence of carbapenem resistance in Hv-Kp strains.

Carbapenems being the last line of drugs available for the effective treatment of multidrug resistant *K. pneumoniae* infections, it has become a major concern as it is evident that we see a dramatic increase in its resistance to the existing drugs and that developing new drugs as a solution cannot be carried out in the same pace [13].

The virulence factors of *Klebsiella pneumoniae* are capsular polysaccharides, siderophores, adhesins, and types 1 and 3 fimbriae. The severity is related to these factors. The virulence factors in ESBLs producing *K. pneumoniae* were biofilm-serum resistant-haemagglutination-BssS-fimH-iss (28%), whereas the non-ESBLs producing isolates were biofilm-haemagglutination-hypermucoviscosity-BssS-fimH (36%). Previous studies have shown a negative association between ESBL production and hypermucoviscosity.

We have found diabetes to be a common predisposing condition in C-Kp group which is contrary to a study conducted by Yawei Zhang et al. [19], where diabetes was found to be closely associated with Hv-Kp infections due to the impaired neutrophil activity caused by poor glycaemic control as explained by Jun-Chung Lin et al. in their study [20].

More than half of the *K. pneumoniae* isolates (58.82%) that caused infections in diabetics were multidrug resistant which was again contrasting the study conducted by Bing Liu et al., showing low antimicrobial resistance in *K. pneumoniae* infections in diabetics [21]. Respiratory disorders like chronic obstructive pulmonary disorder (COPD) and ICU stay were found to be closely associated with Hv-Kp infections. As studies suggest, a majority of Hv-Kp infections arise from the hospital environment [7]. Inhalation of contaminated air with dust particles or contaminated surfaces act as a major source of infection in health care settings and this could be a possible explanation for hospital acquired Hv-Kp infections.

In contrary to the previous studies, the mortality rate was very high in Hv-Kp associated infections (87.5%) than C-Kp associated infections (35.7%) due to multidrug resistance. Even though majority of Hv-Kp isolates were sensitive to carbapenems, the dramatically increasing patterns of antimicrobial resistance are a wakeup call to implement efficient methods to bring a control to this worsening situation.

In our study, 65.2% of hypermucoviscous strains were multidrug resistant. This correlation was found to be statistically significant (*P* < 0.01). Hypermucoviscosity, regulated by a network consisting of genes magA (K1 serotype), rmpA, rmpA2, alone does not have much role in drug resistance, instead, either the resident MDR genes or the acquired plasmid mediated drug resistance could be the reason for its correlation [22, 23]. No significant correlation was found between possession of aerobactin gene and antibiotic resistance.

Emergence of hypermucoviscous C-Kp and multidrug resistant Hv-Kp can be explained by the acquisition of plasmids coding for virulence genes in C-Kp and plasmids coding for multi-drug resistant genes in Hv-Kp via conjugation. This convergence could cause devastating outbreaks with high mortality rates which can be prevented by regularly monitoring the prevalence of Hv-Kp infections in the locality, surveillance of hospital acquired infections, use of rapid diagnostic methods for the detection of C-Kp and Hv-Kp strains, maintenance of hospital and environmental hygiene, proper sanitation and rational use of antibiotics. Many developed countries have seen a decline in antimicrobial resistance after inculcating such precautionary measures and it is the right time for all stake holders to take action in order to tackle this situation [13].

## 6. Conclusion

Our study has recorded the highest percentage of antibiotic resistance in Hv-Kp group compared to previous studies indicating a dramatic increase in multidrug resistant Hv-Kp strains. Our study urges the need to include rapid methods in routine diagnostic tests to identify drug resistance patterns in both the groups. Our study showed a significantly high mortality rate in Hv-Kp group, thus highlighting the importance of regular monitoring of patients, especially those with underlying conditions like diabetes and respiratory disorders and prolonged hospital stay to prevent possible spread of Hv-Kp infections. A combination of hypervirulence and multidrug resistance could result in deadly outbreaks and high mortality rates. Our study helps in identifying the Hv-Kp strains in the clinical samples and determining the antimicrobial susceptibility patterns so as to provide immediate and effective treatment.

## Figures and Tables

**Table 1:** Isolation of *Klebsiella pneumoniae* from various clinical specimen and demographical features of patients included in the study.

Sample type	*Patient details*												Total Isolates
Sex->	Male						Female						
Age group->	<1	1–10	11–20	21–50	51–70	>70	<1	1–10	11–20	21–50	51–70	>70	Total
Blood (central line)	14	0	0	3	8	01	04	0	0	01	03	01	35
Biopsy (deep tissue, etc.)	0	0	0	0	03	01	0	0	0	01	02	0	07
BAL	0	0	0	01	0	02	0	0	0	0	0	0	03
Exudate (pus/Drain/Abscess/Swab)	0	0	0	10	04	01	0	0	0	02	0	01	18
ET suction tip	01	0	0	02	05	03	0	0	0	0	02	0	13
Fluids (CSF/Pleural)	0	0	0	01	01	0	0	0	0	01	01	0	04
Sputum	0	0	0	0	0	01	0	0	0	0	01	02	04
Urine	02	0	0	04	07	06	01	0	02	08	11	04	45
Total	17	0	0	21	28	15	5	0	2	13	20	8	129

**Table 2:** Differentiation of *Klebsiella pneumoniae* isolates into classical (C-Kp) and hypervirulent (Hv-Kp) type based on phenotypic and genotypic markers.

Isolated from	Total	*Virulence marker*		*K. pneumoniae type (%)* ^*∗*^	
		Hyper-mucoviscous	Aerobactin gene	C-Kp	Hv-Kp
Blood (central line)	35	05	04	31 (88.6)	04 (11.4)
Biopsy (deep tissue, etc.)	07	0	0	07 (100.0)	0
BAL	03	0	0	03 (100.0)	0
Exudate (pus/Drain/Abscess/Swab)	18	05	05	13 (72.2)	05 (27.7)
ET suction tip	13	04	02	11 (84.6)	02 (15.4)
Fluids (CSF/Pleural)	04	01	01	03 (75.0)	01 (25.0)
Sputum	04	01	01	03 (75.0)	01 (25.0)
Urine	45	07	05	40 (88.8)	05 (11.1)
Total	129	23	18	111 (86.1)	18 (13.9)

^*∗*^% isolates in each type of infection; classification as Hv-Kp was based on possession of Aerobactin gene.

**Table 3:** Incidence of ESBL production in classical *Klebsiella pneumoniae* (C-Kp) and hypervirulent *Klebsiella pneumoniae* (Hv-Kp) isolates from various types of infections.

Antibiotic	Resistance in %
Amoxiclav	79%
Gentamicin	41%
Amikacin	42%
Ciprofloxacin	45%
Ceftriaxone	67%
Cefaperasone sulbactam	32%
Piperacillin tazobactam	29%
Imipenem	19%
Meropenem	19%

Resistance Rates Aminoglycosides 69/175 (39.4%) Fluoroquinolones 70/175 (40%) 3 rd generation cephalosporins 77/175 (44%) Piperacillin tazobactam 50/175 (29%) Carbapenems 32/175 (18%).

**Table 4:** Multidrug resistance exhibited by classical *Klebsiella pneumoniae* (C-Kp) and hypervirulent *Klebsiella pneumoniae* (Hv-Kp) isolates included in the study.

Infection type	*C-Kp (%)*			*Hv-Kp (%)*		
	One drug	Two	≥ Three	One drug	Two	≥ Three
Sepsis	03	02	25	01	0	02
RTI	01	02	16	04	0	01
UTI	05	04	31	0	02	03
Pyogenic	05	0	13	01	01	02
Total	14 (12.6)	8 (7.2)	85 (76.6)	6 (33.3)^*∗*^	3 (16.6)	8 (44.4)^*∗*^

Five isolates were sensitive to all antibiotics (4 isolates of C−Kp; 1 isolate of Hv−Kp); ^*∗*^Statistically significant; *P* < 0.01.

**Table 5:** Comparison of antibiotic susceptibility patterns of classical *Klebsiella pneumoniae* (C-Kp) and hypervirulent *Klebsiella pneumoniae* (Hv-Kp).

Type of Susceptibility	C-Kp (%) n = 111	Hv-Kp (%) *n* = 18	Significance^*∗*^ (*P* < 0.05)
Non-MDR	26 (23.4)	10 (55.5)	<0.001
MDR	85 (76.6)	08 (44.4)	<0.01
ESBL+	84 (75.7)	08 (44.4)	<0.01

Non—MDR—Resistant to ≤2 classes of antibiotics; MDR—≥3 classes of antibiotics; ESBL+—ESBL producers; ^*∗*^Statistically significant when compared to C−Kp group, in percentage.

**Table 6:** Comparison of Risk factors associated with classical *Klebsiella pneumoniae* (C-Kp) and hypervirulent *Klebsiella pneumoniae* (Hv-Kp) infections and their outcome.

Risk factor	*Type of Isolate*			*Outcome (n-56)*		*Type of Isolate*			*Outcome (n* *=* *8)*	
	C-Kp					Hv-Kp				
	Non-MDR	MDR	ESBL+	Survived	Expired	Non-MDR	MDR	ESBL+	Survived	Expired
Diabetes	07	09	12	12	04	01	0	0	0	01
Cancer	03	05	04	03	05	0	0	0	0	0
CVD	05	06	08	09	02	01	01	0	0	02
CLD	0	02	02	02	0	0	01	01	0	01
CKD	01	05	04	04	02	0	0	0	0	0
RTD	02	07	07	04	05	04	0	01	01	03
HIV	0	01	01	0	01	0	0	0	0	0
AID	0	0	0	0	0	0	0	0	0	0
DTD	01	02	03	02	01	0	0	0	0	0
Total	19	37	41	36	20	6	2	2	1	7^*∗*^

CVD—cardiovascular disease; CLD—Chronic liver disease; CKD—Chronic kidney disease; RTD—Respiratory tract disease; HIV—Human immunodeficiency virus infection; AID–Autoimmune disease; DTD—Digestive tract disease; ^*∗*^mortality rate is statistically significant, with a *P* value of <0.001 when compared to mortality rate of infections with C—Kp.

**Table 7:** Correlation of virulence marker with antimicrobial susceptibility patterns among Hv-Kp isolates (5 hyper-mucoviscous strains which were aerobactin gene negative were also included).

Virulence marker	Non-MDR	MDR	ESBL +
Hyper-viscosity (*n* = 23)	08 (34.7)	15 (65.2)^*∗*^	12 (52.2)
Aerobactin (*n* = 18)	10 (55.6)	8 (44.4)	8 (44.4)

Non−MDR–Resistant to ≤2 classes of antibiotics; MDR—resistant to ≥3 classes of antibiotics; ESBL+—ESBL producers; ^*∗*^Incidence higher (statistically significant) when compared to non−MDR isolates.

**Table 8:** Infections caused by classical and Hv-Kp.

Infection type^*∗*^	C-Kp (%)			Hv-Kp (%)		
	Non-ESBL	ESBL	Total	Non-ESBL	ESBL	Total
Sepsis	04 (12.9)	27 (87.1)	31	03 (75.0)	01 (25.0)	04
RTI	04 (20.0)	16 (80.0)	20	03 (60.0)	02 (40.0)	05
UTI	13 (32.5)	27 (67.5)	40	03 (60.0)	02 (40.0)	05
Pyogenic	08 (40.0)	12 (60.0)	20	02 (50.0)	02 (50.0)	04
Total	29	82	111	11	07	18

## Data Availability

All datasets generated or analysed during this study are included in the manuscript.
